# Can Phonemic Verbal Fluency Be Used to Predict Alzheimer’s Disease?

**DOI:** 10.3390/neurosci5040036

**Published:** 2024-11-04

**Authors:** Sara García-González

**Affiliations:** School of Education and Psychology, University of Navarra, 31009 Pamplona, Spain; sggonzalez@unav.es

**Keywords:** Alzheimer’s disease, cognitive markers, language and verbal fluency

## Abstract

Background: Among the cognitive markers, the deterioration of semantic and phonemic verbal fluency seems to be an early indicator of Alzheimer’s disease (AD). The aims of this study are (1) to evaluate both types of verbal fluency in the early stages of AD in order to know which of them deteriorates earlier and (2) to investigate if verbal fluency tasks can help to differentiate between patients with Mild Cognitive Impairment (MCI) who will progress to AD two years later (progress) and those who will not (non-progress). Method: A verbal fluency task was administered to 25 patients with MCI and their respective control subjects. All patients underwent a neuropsychological evaluation twice in order for us to follow up on their global cognitive status. The second time, eight of them converted to AD. Results: On the one hand, phonemic verbal fluency deteriorates earlier than semantic verbal fluency in MCI patients; on the other hand, although we found statistically significant differences between patients with MCI and AD in both types of fluency tasks, none were found when comparing the performance of progress and non-progress. Conclusions: These results point to a greater impairment in phonemic verbal fluency in MCI patients and its potential capacity to predict conversion to AD.

## 1. Introduction

Alzheimer’s disease (AD) is the most common form of dementia in the elderly, accounting for more than 60% of all cases [1]. It is characterized by a slow onset and progressive cognitive decline that affects higher functions such as language, personality, memory, visuospatial perception and knowledge [2]. Consequently, this neurodegenerative disease is a serious problem that decreases the quality of life of patients and their relatives.

The stage at which AD is diagnosed influences the recommended therapy, patient and family counseling and the approach to long-term care [3]. Therefore, the challenge in AD diagnosis today is in diagnosing patients before the cognitive deficits have reached the threshold of dementia, that is to say, in its prodromal stage. Several terms have been used to describe this pre-dementia phase. The most popular one is Mild Cognitive Impairment (MCI), proposed by Petersen et al. in the late 1990s [4,5].

In the last decade, there has been unprecedented growth in scientific knowledge about early diagnosis, especially regarding biomarkers and neuroimaging techniques. However, in a clinical context, the diagnosis of AD is mainly based on neuropsychological testing. The best-known cognitive markers of this pathology are as follows: (1) deterioration of episodic memory, which does not improve even with cues and involves the presence of numerous intrusions and perseverations; (2) reduced social and occupational performance; (3) anosognosia; (4) anomie or difficulty in naming; (5) spatial disorientation in unfamiliar places; (6) deficits in visual processing speed and in selective and divided attention; (7) communicative difficulties; and (8) impaired semantic memory [6,7,8,9]. Among the cognitive markers mentioned, it is worth highlighting naming problems, which is one of the earliest and most notorious manifestations of language impairment. Naming problems are usually measured by verbal fluency (VF) tasks, in which the subject is asked to produce as many words as possible in a given time [10,11]. The two forms of verbal fluency that are commonly assessed are phonemic—related to the retrieval of words that begin with a certain letter or phoneme—and semantic—related to the ability to produce series of words that belong to a semantic category such as animals or fruits [11]. It also has to be said that these tasks are related not only to the activation of processes linked to lexical access, but also to executive processes—which are also altered with age, especially in cognitive impairment processes [12,13]. Therefore, when a person performs a verbal fluency task, the areas of the brain responsible for the aforementioned cognitive processes should be activated. More specifically, it is known that phonemic verbal fluency (PVF) mainly involves the activation of temporal regions, which reflects executive functioning, and semantic verbal fluency (SVF) primarily involves the activation of frontal regions and is assumed to reflect semantic knowledge.

Several studies have found SVF to be a very sensitive assessment of semantic impairment at a very early stage of AD [8,14,15,16,17], but this is not a universal finding. On the one hand, Whels et al. [18,19] did not find statistically significant differences between patients with mild AD and healthy elderly control subjects upon performing an SVF task, and Albert et al. [20] reported normal performance in naming and category fluency in a group of patients categorized as 0.5 on the clinical dementia rating (CDR) scale [21]. On the other hand, Goñi et al. [22] found that people with Alzheimer’s disease performed worse than the control group in a PVF task, so they deduced that it deteriorates before SVF. In this sense, there seems to be an unresolved debate about the type of fluency that deteriorates earlier in AD. For this reason, the aims of this research are, on the one hand, to evaluate both types of VF in people with early stages of Alzheimer’s disease in order to know which of them deteriorates earlier, and, on the other hand, to investigate whether either of them predicts the evolution of MCI to AD.

## 2. Materials and Methods

### 2.1. Participants

Twenty-five MCI patients and their respective control subjects, matched by age, sex and educational level, took part in this study (see Table 1). Since there is a broad consensus on the influence of educational level, the participants were separated into three groups: Low (from 0 to 4 years), Medium (from 5 to 10 years), and High (more than 10 years) educational level. It was decided not to separate by gender, as there seems to be an unresolved debate about the influence of gender on verbal fluency task performance [14].

The experimental group was recruited at the Neurology Unit of the University Hospital of Cabueñes (Asturias, Spain). All of the participants were administered a neuropsychological battery that included screening tests and tests assessing memory, language, executive functions and visuospatial skills as part of their annual monitoring. The control group was enrolled from cultural centers, day centers and the University Program for the Elderly of the University of Oviedo.

All participants were administered a screening test and a verbal fluency task. The results of the former served as selection criteria, while the results of the latter were used to compare the performance of the control subjects versus the performance of the patients with MCI. The neuropsychological data of both groups are shown in Table 1.

Two years after the first evaluation, the MCI patients were followed up and eight of them had converted to probable AD (MCI-AD).

The inclusion criteria used at the University Hospital of Cabueñes for MCI patients were as follows: (1) objective memory impairment on neuropsychological evaluation; (2) normal activities of daily living; (3) an absence of dementia; (4) an absence of depression; (5) no presence of other psychiatric diseases, epilepsy or drug addiction, and (6) current or previous uncontrolled systemic diseases or recent traumatic injuries. Medical history and neuroimaging tests were also reviewed for all patients. For their part, the control subjects had to meet two conditions: they had to (1) be over 65 years of age and (2) have an MMSE score equal to or higher than 26. The exclusion criterion for both groups was the presentation of any psychiatric, neurological (except MCI in the case of the experimental group) or medical diseases that could interfere with the performance of the tests in this study.

The NIA-AA criteria (2011), proposed by the National Institute of Aging (NIA) and the Alzheimer’s Association (AA), were used to identify those MCI patients who converted to probable AD [23].

All participants signed their informed consent after being informed of the study characteristics by their regular neurologist or psychologist. It should also be mentioned that this research complied with the standards established by the National Health Council on research involving human subjects, and the study was approved by the Clinical Research Ethical Committee of Asturias.

### 2.2. Material

Two screening tests were applied to describe the general cognitive functioning of the participants: the Spanish adaptation of the Mini-Mental State Examination (MMSE) [24] and the Spanish version of the Montreal Cognitive Assessment (MoCA) [25]. The former is the most commonly used screening method in the assessment of the severity of dementia in both the clinical and research fields; but the latter is better at detecting MCI among patients over 60 years of age than the MMSE [26].

After cognitive screening, verbal fluency was assessed. First, as part of the MoCA, participants were asked to generate words that start with the letter P, but neither names of people nor names of cities were allowed, to assess PVF. Then, participants were asked to produce words in the animal category to assess SVF. In each trial, they were told to generate as many words as possible within a minute.

In the phonemic verbal fluency task, the choice of the letter depends on the language of the participants. In this case, the letter “P” was selected since it is the most informative [27].

### 2.3. Procedure

The subjects were evaluated in one single session in an individual room in order to avoid possible distractions that could bias the results.

The neuropsychological assessments were conducted by a psychologist who was attentive to the behavior of the subjects under testing. Any evidence of unexpected behavioral responses during the procedure was registered and taken into account.

The questionnaires were administered in the following order: (1) MMSE; (2) MOCA; and (3) verbal fluency tasks.

## 3. Results

The results obtained were statistically analyzed using the statistical software package IBM SPSS Statistics 21 (Chicago, IL, USA).

Following the observation of the results obtained in the verbal fluency test in both the control and experimental groups, it is observed that the performance of the former is higher (see Table 1). So, the next step was to check if this difference was statistically significant or not.

The individual test scores for verbal fluency task were transformed into Z-scores based on a larger sample’s mean and standard deviation (see Table 1). Next, to determine whether it is SVF or PVF that deteriorates earlier in subjects with MCI, a Student’s *t*-test was performed (see Table 2).

As can be seen in Table 2, there are statistically significant differences between the experimental and control groups in PVF (t = −2.30 *p* = 0.026) but not in SVF performance (t = −1.56 *p* = 0.124). Hence, it seems that PVF deteriorates earlier.

Finally, to investigate if verbal fluency tasks can help to predict the conversion from MCI to probable AD two years later, we analyzed the differences between the eight patients who converted to AD (MCI-AD) and the seventeen who did not (MCI-nonAD) at the time in which they all maintained their original diagnosis (see Table 3). Due to the small sample size and non-compliance with the normality criterion, a Mann–Whitney U test was conducted. The descriptive statistics are provided in Table 4.

It is worth noting the age difference between both groups of MCI patients: some of them developed symptoms at an early age, while others developed them at an older age. However, the difference of almost 3 years between both groups is not statistically significant.

After analyzing the data, no statistically significant differences were found between PVF and SVF in both groups. Nevertheless, if we compare the VF task performance between the eight patients with the diagnosis of probable AD (AD group)—two years after the first evaluation—and the seventeen who maintained the original diagnosis of MCI (MCI group), we find statistically significant differences between PVF and SVF in both groups (see Table 5).

Taking all these results together, it could be that either a larger sample size would allow statistically significant differences to be obtained, or that the deterioration of PVF could help predict the conversion of MCI to probable AD.

## 4. Discussion

Efforts toward the earlier detection of AD face significant challenges such as improving the assessment of the earliest symptoms. Memory impairment is typically the most common manifestation of early AD, but growing evidence suggests that impairments to other cognitive domains such language dysfunction begin several years before the onset of dementia, suggesting that this could be a possible prognostic marker [9,28] that should be taken into account in neuropsychological assessments.

Language impairment initially affects verbal fluency and naming. Regarding fluency, although it is well known that patients with AD begin to produce a lower number of words in both semantic and phonemic verbal fluency compared with age-and education-matched elderly control subjects, controversy exits regarding the type of fluency measure that best discriminates patients with early AD from healthy elderly individuals. In this sense, our results show that the experimental group performed worse than the control group in both types of fluency. What is more, the SVF scores of the experimental group were slightly higher than those of the PVF group, and statistically significant differences were found.

These data are consistent with those of authors such as Goñi et al., Montañés, and Comesaña and Coni [22,27,29]. They suggest that SVF is better preserved over time than PVF due to the executive impairment of AD patients. SVF tasks are considered to be easier to perform than PVF tasks because the retrieval of words beginning with a certain letter involves searching a high subset number of categories and requires higher executive functioning performance. In fact, one of the least-known cognitive symptoms of Alzheimer’s disease is impaired executive function, present from early stages, which hinders the proper functioning of working memory, attention, inhibition and decision making—among other functions.

Regarding assessing the progression of MCI to AD, although we found statistically significant differences between patients with MCI and AD in both types of fluency tasks, they were not found when comparing the performance of subjects with MCI and those with MCI who converted to AD two years later. Similarly, Vaughan et al. [30] could not differentiate between progress and non-progress using individual fluency measures. Tracking progression from MCI to AD requires not only accurate diagnosis, but also cognitive measures sensitive to change over time [31]. In this sense, Vaughan et al. [30] proposed using discrepancy scores at baseline to discriminate between patients with MCI who remained stable and those with MCI who converted to AD after two years. To calculate discrepancy scores for each participant, they subtracted the phonemic fluency results from the semantic ones. They concluded that MCI patients with a phonemic advantage at initial evaluation have a greater likelihood of progressing to AD. But the truth is that other studies of relative semantic–phonemic discrepancies in MCI have yielded discordant results. Therefore, future research is needed to determine the specificity of such findings to AD and their utility in serial assessments over time.

The current study is subject to certain limitations, in particular, the small sample size, which makes it difficult to generalize the results and determine the selection of statistical tests (for example, the use of the Mann–Whitney U test instead of the *t*-test). More participants who meet the required criteria will be recruited in the future to mitigate this limitation. Secondly, it is known that there is great variability in fluency results depending on the fluency task used. This study focuses only on the “letter p” and the category “animals” because they are the most used in clinical practice. Although using combined categories is more reliable than using individual letters or categories, the results obtained remain clinically applicable because “letter p” fluency is part of the Montreal Cognitive Assessment, a commonly administered assessment. Cognitive screening and animal fluency can be easily added to any assessment protocol. Finally, it is worth mentioning that some researchers report that semantic fluency tasks that use large categories (e.g., animals) contain more disease-associated variance but are more sensitive for detecting AD. [32]. To counteract this variability, Z scores were used in the statistical analyses (see Table 2, Table 4 and Table 5). Finally, it is also worth mentioning the strengths of this study. First, the diagnosis was undertaken by a specialized neurologist using standardized neuropsychological assessments and the latest published AD diagnostic criteria [23]. Second and last, this study promotes more research on this topic that continues to generate great controversy.

## 5. Conclusions

Due to the high number of people diagnosed with Alzheimer’s disease and the lack of a cure for the disease, there is an imperative need for early detection. Although much research has been conducted to identify biomarkers related to AD, neuropsychological evaluation remains essential for diagnosis. Despite memory dysfunction being the most common manifestation of early AD, some cases first present with executive, language or visuospatial disturbances. Our results support the idea that there are already linguistic alterations in the early stages. Tests of verbal fluency provide good discrimination between persons with normal cognitive function compared to those with Mild Cognitive Impairment. Thus, in order to make an early diagnosis, verbal fluency should be taken into account, especially phonemic verbal fluency, which is less preserved over time than semantic verbal fluency. Additionally, further longitudinal studies are needed to clarify whether this may be clinically relevant in predicting progression of MCI to AD.

## Figures and Tables

**Table 1:** Demographic and neuropsychological data.

Variables	Control Group	Experimental Group
N	25	25
Age (Mean ± SD)	73.68 ± 5.12	74.60 ± 5.12
Education (L/M/H)	7/12/6	7/12/6
Gender (M/F)	15/10	15/10
MMSE (Mean ± SD)	28.96 ± 1.17	26.13 ± 2.50
MoCA (Mean ± SD)	26.44 ± 2.40	20.10 ± 3.95
PVF (Mean ± SD)	14.36 ± 3.47	12.08 ± 3.53
SVF (Mean ± SD)	16.76 ± 4.30	14.88 ± 4.20

N: sample number; SD: standard deviation; L: Low; M: Medium; H: High; M: male; F: female; MMSE: Mini-Mental State Examination; MoCA: Montreal Cognitive Assessment; PVF: phonemic verbal fluency; SVF: semantic verbal fluency.

**Table 2:** A comparison of the PVF and SVF performance of the MCI and control groups (Z-scores).

VF	Group	N	Mean	SD	t	*p*
ZPVF	MCI MCI-C	25 25	−0.18 0.38	±0.88 ±0.86	−2.30	0.026 *
ZSVF	MCI MCI-C	25 25	−0.34 0.35	±0.87 ±0.89	−1.56	0.124

ZPVF: Z-score of phonemic verbal fluency; ZSVF: Z-score of semantic verbal fluency. * *p* < 0.05

**Table 3:** A comparison of the PVF and SVF performance of the MCI-AD and MCI-nonAD groups (Z-scores).

VF	Group	Mean ± SD	U	*p*
ZPVF	MCI-nonAD MCI-AD	−0.01 ± 0.72 −0.55 ± 1.12	38.50	0.086
ZSVF	MCI-nonAD MCI-AD	−0.07 ± 0.87 −0.27 ± 0.89	56.50	0.511

**Table 4:** Demographic data and Z-scores of PVF and SVF.

Variables	MCI-AD	MCI-NonAD
N	8	17
Age (Mean ± SD)	72.38 ± 4.34	75.65 ± 5.23
Education (L/M/H)	2/4/4	5/8/4
Gender (M/F)	4/4	11/6
MMSE	24.75 ± 1.83	26.94 ± 2.51
MoCA	17.25 ± 2.60	21.41 ± 3.81

MMSE: Mini-Mental State Examination; MoCA: Montreal Cognitive Assessment.

**Table 5:** A comparison of the PVF and SVF performance of the MCI and AD groups (Z-scores).

VF	Group	Mean ± SD	U	*p*
ZPVF	MCI AD	−0.01 ± 0.72 −1.26 ± 0.84	17.50	0.002 *
ZSVF	MCI AD	−0.07 ± 0.87 −1.38 ± 0.53	8.50	0.000 *

* *p* < 0.05.
